# A Multi-Locus Association Model Framework for Nested Association Mapping With Discriminating QTL Effects in Various Subpopulations

**DOI:** 10.3389/fgene.2020.590012

**Published:** 2021-01-18

**Authors:** Suhong Bu, Weiren Wu, Yuan-Ming Zhang

**Affiliations:** ^1^College of Agriculture, South China Agricultural University, Guangzhou, China; ^2^Key Laboratory of Genetics, Breeding and Multiple Utilization of Crops, Ministry of Education, Fujian Agriculture and Forestry University, Fuzhou, China; ^3^Crop Information Center, College of Plant Science and Technology, Huazhong Agricultural University, Wuhan, China

**Keywords:** nested association mapping (NAM), multi-locus association model, joint-family, subpopulation, maize

## Abstract

Nested association mapping (NAM) has been an invaluable approach for plant genetics community and can dissect the genetic architecture of complex traits. As the most popular NAM analysis strategy, joint multifamily mapping can combine all information from diverse genetic backgrounds and increase population size. However, it is influenced by the genetic heterogeneity of quantitative trait locus (QTL) across various subpopulations. Multi-locus association mapping has been proven to be powerful in many cases of QTL mapping and genome-wide association studies. Therefore, we developed a multi-locus association model of multiple families in the NAM population, which could discriminate the effects of QTLs in all subpopulations. A series of simulations with a real maize NAM genomic data were implemented. The results demonstrated that the new method improves the statistical power in QTL detection and the accuracy in QTL effect estimation. The new approach, along with single-family linkage mapping, was used to identify QTLs for three flowering time traits in the maize NAM population. As a result, most QTLs detected in single family linkage mapping were identified by the new method. In addition, the new method also mapped some new QTLs with small effects, although their functions need to be identified in the future.

## Introduction

Association mapping of large genetically diverse population has advantages over quantitative trait locus (QTL) mapping of biparental segregation population, such as the ability to access multiple gene alleles and higher mapping resolution (Zhang et al., 2005; Korte and Farlow, 2013). This is because the former carries more recombination breakpoints in history. However, the genetic structure of genome-wide association study (GWAS) population leads to high false positive rates (FPRs; Yu and Buckler, 2006). Moreover, low allele frequencies confer low statistical power (Rafalski, 2010). To address these issues, multiparental population or next-generation mapping populations, such as nested association mapping (NAM) and multiparent advanced generation intercross (MAGIC), were proposed (Cavanagh et al., 2008; Yu et al., 2008; Morrell et al., 2012). It was proved to have sufficient power and resolution to detect genomic associations for plant complex traits.

The NAM population was a special kind of multiparental panel, which was first proposed in maize (Yu et al., 2008). They crossed 25 representative lines with homozygous B73 line to generate 25 populations that consisted of 5,000 recombinant inbred lines (RILs; McMullen et al., 2009) and demonstrated that the NAM population method was powerful in dissecting the genetic architecture of complex traits, including flowering time, leaf architecture, stalk strength, and plant height (Buckler et al., 2009; Tian et al., 2011; Peiffer et al., 2013, 2014; Li et al., 2016). This initial success prompted the development of the NAM population in other crops, such as rice, wheat, barley, soybean, and sorghum (Maurer et al., 2015; Schmutzer et al., 2015; Bajgain et al., 2016; Bouchet et al., 2017; Fragoso et al., 2017; Song et al., 2017). Taking a wide view of all NAM methods applied in previous studies, they were prone to joint linkage across all subpopulations over single population mapping, as single population analysis has far less power and accuracy than joint mapping, although it will not position QTL inaccurately (Buckler et al., 2009). However, these approaches did not take into account the potential difference of QTL effects across families.

**FIGURE 1:**
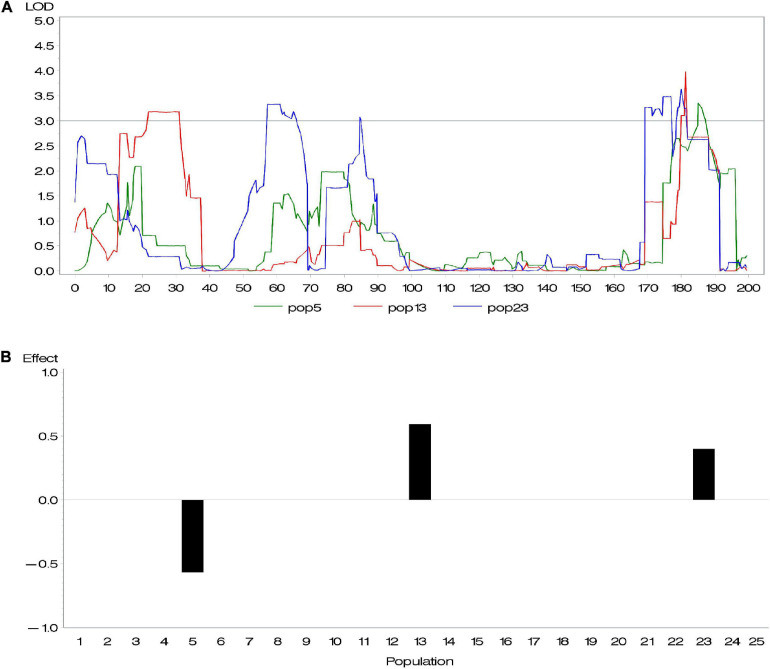
**(A)** An overlapped quantitative trait locus (QTL) identified in subpopulations 5, 13, and 23 and **(B)** its effects.

Genetic heterogeneity from different parents is likely to contribute to potential diversity of genetic architecture across subpopulations. Buckler et al. (2009) investigated the difference of allelic effects across different founder lines and demonstrated that the difference of QTL effects across subpopulations is related to latitudinal variation. Given that this diversity exists, the above methods, considering all QTL with same effects across all subpopulations, are not appropriate. To address this issue, we conducted a series of composite interval mapping (CIM; Zeng, 1994) for each RIL population in the maize NAM population. The results showed that QTLs detected in different subpopulations did not share either the same position or effect (Supplementary Table 1). For instance, different RIL populations might detect different QTLs; even if QTLs were detected across more than one population, these QTLs could rarely share the same effect. Figure 1 shows an example of overlapped QTL. Within a distance of 10 cM, there were three QTLs identified in various subpopulations and having quite different effects. Because their peaks were very close, these QTLs were treated as an overlapped QTL. The results confirmed our suspicion. In association mapping in multiparental population, therefore, it is necessary to discriminate QTL effects in various subpopulations.

In this study, we proposed a speculation that QTL shared across multiple subpopulations of NAM has different effects in genetic mapping model. It was a specialty for the NAM design and also other similar multiple populations from multiple parents. A multi-locus association model was introduced to dissect the genetic basis of complex traits. In this kind of statistical model, variables involved are extremely colossal when single-nucleotide polymorphism (SNP) makers are numerous. Thus, we suggested a new matrix transform approach to address the problem of super-high dimensions. A series of Monte Carlo simulation experiments based on NAM marker data were performed to demonstrate the performance of this new method. Additionally, the validated approach was applied in genetic analysis for three flowering time traits in maize.

## Materials and Methods

### NAM Population

We used the maize NAM population data (Buckler et al., 2009) from the Panzea website.^1^ The NAM population consists of 4,699 RILs derived from the crosses between 25 diverse lines and the common parent B73. All the RILs from each cross were considered as a subpopulation. A total of 1,106 SNP markers were genotyped for each RIL, covering a genetic map of 1,400 cM and one marker every 1.3 cM on average. The best linear unbiased predictions (BLUPs) of three flowering time traits, including days to anthesis (DA, male flowering), days to silking (DS, female flowering), and anthesis-silking interval (ASI), were used as the phenotypic data in following analysis.

### Genetic Model

Suppose that a general NAM design is as follows: *k* selected founder lines are crossed to a common parent, followed by selfing to generate *k* segregation F_2_ populations, and each F_2_ population are used to generate a half-sib subpopulation composed of *n* RILs by selfing for multiple generations. The phenotypic value of a quantitative trait may be described by the following model:

(1) $$Y=\lambda \,\mu +\sum_{i=1}^{q}\sum_{j=1}^{k}X_{i\,j}\,\beta_{i\,j}+\varepsilon$$

where *Y* = (*y*_1_,*y*_2_,…*y*_*kn*_)′; μ is a 25 × 1 matrix of covariant components; each element represents one subpopulation phenotype mean; λ is the *kn*×25 indicator matrix relating to each subpopulation; *q* is the number of QTL associated with interested trait; *k* is the number of sub-populations; and ε is the vector of residual error with a *N*(0,σ^2^) distribution. β_*ij*_ represents the additive effect of the *i*th QTL in the *jt*h subpopulation. Namely, we gave *k* effects for one QTL across the *k* subpopulations. *X*_*ij*_is a *kn*×1incidence vector of the *i*th QTL in the *j*th subpopulation. In this incidence vector, the *n* elements corresponding to the *j*th subpopulation are coded (−1, 1), representing the genotype of SNP (AA and aa), and the other (*k*−1)*n* elements are assigned 0, suggesting the absence of this QTL effect in other subpopulations. In multi-locus model, all available SNPs are considered as candidate QTL to be incorporated in the genetic model. Thus, the numerous variables in the model from huge number of SNPs and many subpopulations make a big burden for computing.

In order to relieve the computing burden, the dimensions of incidence matrix need to be reduced. Thus, we proposed a strategy to achieve dimension reduction and also make sure that the incidence matrix still involves different subpopulation information. In this method, *k* column original incidence vectors, corresponding to one QTL in all subpopulations, are emerged into one column vector. Here is the process: as for an SNP, we first calculate the main effect of each genotype in all subpopulations, respectively, $\omega_{i\,j}={\bar{y}}_{i\,j}-\bar{y}$, where *i* = 1,…,*k*, *j* = 1,2 respects AA and aa. Thus, a vector ω, consisted of 2*k* indicators, is obtained and then sorted. Next, we recode the genotypes across all subpopulations according to their effects’ order and obtain a transformed incidence vector *Z*_*i*_ for a given QTL (SNP) (Lü et al., 2011). In addition, *Z*_*i*_ could be also transformed according to segmented ω. The genetic model is transformed as:

(2) $$Y=\lambda \,\mu +\sum_{i=1}^{q}Z_{i}\,\gamma_{i}+\varepsilon$$

where *Z*_*i*_ is a kn× 1 incidence vector of the *i*th QTL in all subpopulations, and γ_*i*_ is the corresponding effect of the *i*th putative QTL.

### Multi-Locus Association Analysis

To select, estimate, and validate loci associated with interested trait, we proposed a multi-locus association of two-stage processes. Based on the genetic model (2), genome SNPs scanning needs to further select, estimate, and validate SNPs associated with given trait. We proposed the following two-stage selection process to screen. In the first stage, shrinkage estimate algorithm was used to estimate the additive effect of SNPs, and all SNPs with $t_{i}=\left|{\hat{\gamma }}_{j}\,/\,{\hat{\sigma }}_{j}\right|>10^{-4}$ are picked up. Considering stability, effectiveness, and computing time, we adopted the empirical Bayes (E-Bayes) method (Xu, 2010). Compared with other shrinkage estimation (Zhang and Xu, 2005; Yi and Banerjee, 2009; Feng et al., 2013), E-Bayes provides a more robust shrinkage that the large effect subjects are shrunk to virtually no shrinkage while small effects to zero, so that nonsignificant SNP is estimated toward zero. Simulation studies showed that the E-Bayes is predominant compared with other shrinkage estimation methods in terms of small mean squares error (Xu, 2010). For the technical details of the method, refer to the original study by Xu (2010). The method is briefly described here.

The parameters β and σ^2^ are always included in the model; the uniform prior is assigned to the two parameters: *P*(β)∝1 and *P*(σ^2^)∝1 (Zhang and Xu, 2005). We adopt the normal prior for each of the genetic effects (γ_*k*_) in model (2): $P\,\left(\gamma_{k}\right)\propto N\,\left(0,\sigma_{k}^{2}\right)$. The scaled inverse χ^2^ prior distribution is further assigned to $\sigma_{k}^{2}$: $P\,\left(\sigma_{k}^{2}\right)=\mathrm{Inv}-\chi^{2}\,\left(\sigma_{k}^{2}|\tau ,\omega \right)\propto {\left(\sigma_{k}^{2}\right)}^{-\frac{\tau +2}{2}}\,\exp\left(-\omega \,/\,2\,\sigma_{k}^{2}\right)$ (Xu, 2010). Clearly, *Y* in model (2) follows a multivariate normal distribution with mean μ = *X*β and variance–covariance $V=\sum_{k}Z_{k}\,Z_{k}^{T}\,\sigma_{k}^{2}+I\,\sigma^{2}$. Let θ = (β,γ,σ^2^). Therefore, the main steps for parameter estimation are described as below.

Step (0): Let *ξ* = (τ,ω) = (0,0), $\hat{\beta }={\left(X^{T}\,X\right)}^{-1}\,X^{T}\,Y$, ${\hat{\sigma }}^{2}={\left(Y-X\,\hat{\beta }\right)}^{T}\,\left(Y-X\,\hat{\beta }\right)\,/\,n$, and γ_*k*_ and $\sigma_{k}^{2}$ were initialized (*k* = 1,2,…,2*m*^2^);

Steps (1): Using $E\,\left(\gamma_{k}\right)=\sigma_{k}^{2}\,Z_{k}^{T}\,V^{-1}\,\left(y-X\,\beta \right)$ and $v\,a\,r\,\left(\gamma_{k}\right)=I\,\sigma_{k}^{2}-\sigma_{k}^{2}\,Z_{k}^{T}\,V^{-1}\,Z_{k}\,\sigma_{k}^{2}$, $E\,\left(\gamma_{k}^{T}\,\gamma_{k}\right)$ was estimated by $E\,\left(\gamma_{k}^{T}\right)\,E\,\left(\gamma_{k}\right)+t\,r\,\left[v\,a\,r\,\left(\gamma_{k}\right)\right]$. This is the E-step;

Step (2): update β, σ^2^ and $\sigma_{k}^{2}$: $\sigma_{k}^{2}=\left[E\,\left(\gamma_{k}^{T}\,\gamma_{k}\right)+\omega \right]\,/\,\left(\tau +2+1\right)$,β = (*X*^*T*^*V*^−1^*X*)^−1^*X*^*T*^*V*^−1^*y*, and $\sigma^{2}={\left(y-X\,\beta \right)}^{T}\,\left[y-X\,\beta -\sum_{k=1}^{m}Z_{k}\,E\,\left(\gamma_{k}\right)\right]\,/\,n$. This is the M-step;

Step (3): Repeat the E-step and the M-step until convergence is reached.

After the reduction in dimension in the first stage, maximum likelihood method could be used to reanalyze the reduced model and perform the likelihood ratio test (LRT) in the second stage. LRT was aimed to decide the inclusion and retention of a SNP in the model based on LR score:

$$L\,R_{j}=-2\,\ln\left[L\,\left(\theta_{-j}\right)\,/\,L\,\left(\theta \right)\right]$$

where θ is the parameter vector in the reduced genetic model; θ_*–j*_ is the parameter vector in θ excluding the currently tested genetic effect $\hat{\gamma }$. *L*(θ) and *L*(θ_−*j*_) are the maximum likelihood function for θ and θ_*–j*_, respectively. If *LR*_*j*_exceeds one given threshold, then it indicates that this SNP could significantly improve model fit. For simplicity, we suggested an alternative statistical parameter *LOD* = *LR*_*j*_/4.61 and 3.0 as the critical value in our association mapping process.

### Monte Carlo Simulation Design

For ease of computation, only few subpopulation data from the maize NAM population including 100 SNP markers from chromosome 1 were used to perform the simulation experiments. The length of chromosome segment was 153.4 cM. We investigated four simulation scenarios, and each simulation had 10 assumed QTLs locating at the given chromosome segment evenly. All the QTL were overlapped with the markers and listed in Supplementary Table 2.

In the first scenario, the effect of QTL heritability on the new method was assessed in five populations with 964 RILs. We assumed 10 QTLs in each of three simulations. The size (or heritability, $h_{i}^{2}$) of each QTL, the proportion of total phenotypic variance explained by the QTL, was all set to 0.03 in the first simulation, 0.05 in the second simulation, and 0.08 in the third simulation. We supposed that each of 10 QTLs had different fixed effects α_*i*_ (*i* = 1, 2, …, 5) among the five populations, and∑_*i*_α_*i*_ = 0. The breeding value of each RIL *i* from population *k* was calculated as*a*_*ki*_ = ∑_*j*_*X*_*j*_α_*kij*_ (*j* = 1, 2, …, 10), and the phenotypic value *y*_*ki*_ = *a*_*ki*_ + *e*_*ki*_,*e*_*ki*_was a residual effect sampled from a normal distribution with mean 0 and variance $\sigma_{e}^{2}=1$. The additive genetic variance of the *i*th QTL,$\sigma_{a\,i}^{2}$, was calculated from $\sigma_{a\,i}^{2}=h_{i}^{2}\,\sigma_{e}^{2}\,/\,\left(1-\sum h_{i}^{2}\right)$. Then, the QTL effects within a given populations were calculated by relating $\sigma_{a\,i}^{2}$ to the allelic frequencies and effects.

In the second scenario, we evaluated the effect of sample size on the new method by setting the sample size as 400 (four subpopulations each with 100 RILs), 600 (six subpopulations each with 100 RILs), and 800 (eight subpopulations each with 100 RILs). Each QTL size was set as 0.07. Other parameters were the same as those in the first scenario.

In the third scenario, we explored the feasibility of a new method on random-effect QTLs. Ten assumed QTLs have the same positions with those in the former two scenarios. The first five QTLs shared a fixed effect (1.5) across all subpopulations. For the *j*th of the latter five QTLs, five effects α_*ij*_(*i* = 1, 2, …, 5) were randomly sampled from multivariate normal distribution with mean 1.5 and variance–covariance structure

$$\Sigma =\left[\begin{array}{ccccc} 1 & \rho & \rho^{2} & \rho^{3} & \rho^{4}\\ \rho & 1 & \rho & \rho^{2} & \rho^{3}\\ \rho^{2} & \rho & 1 & \rho & \rho^{2}\\ \rho^{3} & \rho^{2} & \rho & 1 & \rho \\ \rho^{4} & \rho^{3} & \rho^{2} & \rho & 1 \end{array}\right].\rho$$

was the correlation of QTL effects between two nearest populations and set with two levels (ρ = 0.2 and ρ = 0.8). The proportion of nongenetic variance $\sigma_{e\,k}^{2}$ to total additive genetic variance $\sigma_{a\,k}^{2}$ in population *k* was related to a magnitude of heritability for a trait. In this scenario, the total heritability was set to 0.6.

## Results

### Mapping QTLs for DA, DS, and ASI in Single Maize NAM Subpopulation via the CIM Method

We performed CIM mapping, implemented by Windows QTL Cartographer V2.5,^2^ for DA, DS, and ASI in each NAM subpopulation. For DA in maize, approximately five to six QTLs were detected in each subpopulation. A total of 137 QTLs were identified, with a LOD threshold of 3. Among the 137 QTLs, 10 QTLs clusters (defined with more than five QTLs within a 30 cM interval) were dispersed across 10 chromosomes (Supplementary Table 1 and Supplementary Figure 1). We found 28 overlapped loci (where more than two QTLs from various subpopulations totally or partially overlapped), whereas no same QTL was found across all 25 subpopulations. For most of those overlapped loci, one QTL contributed different effects in different subpopulations. Figure 1 gave an example of an overlapped QTL. Three subpopulations (5, 13, and 23) detected one QTL in a small 177–189 cM interval on chromosome 1 (Figure 1A), where their effects in the three subpopulations are -0.57, 0.40, and 0.60, respectively (Figure 1B). Yet, there were few overlapped QTLs with similar effects across various subpopulations (Supplementary Table 1). In addition, we found a relatively large proportion of total phenotypic variance explained by all the QTLs, such as 66.4% for DA, 74.6% for DS, and 94.4% for ASI.

### Simulation Results

#### Effect of QTL Size on Mapping QTL

**FIGURE 2:**
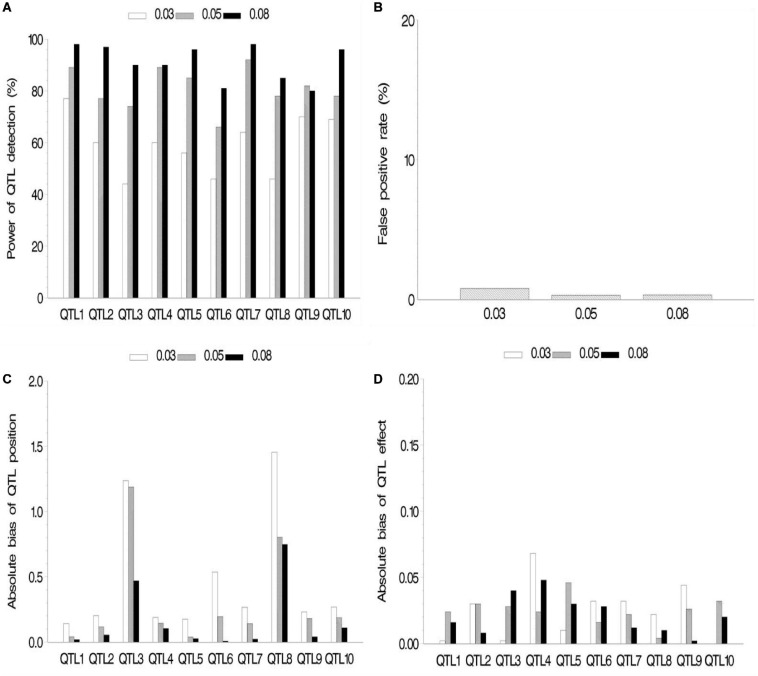
Effect of quantitative trait locus (QTL) heritability on the new method. **(A)** Power of QTL detection, **(B)** false positive rate, **(C)** average of absolute bias between estimated and true positions, and **(D)** average of absolute bias between estimated and true effects.

In the first simulation experiment, the effect of QTL size on mapping QTL in the maize NAM population was evaluated. QTL size was set as 3, 5, and 8%. Ten assumed QTLs were uniformly distributed across the genome in the three cases. Each sample was analyzed by the new method, and the results are shown in Figure 2 and Supplementary Table 2.1. The average power for 10 assumed QTLs in each case was 59.2, 81, and 91.1% for the QTL sizes of 3, 5, and 8%, respectively, indicating the increase in average power of all the 10 assumed QTLs with the increase of QTL size (Figure 2A). The FPR was less in both 5 and 8% cases than in 3% case (Figure 2B). The bias of QTL position estimate was relatively low, and it had a negative correlation with QTL size (Figure 2C). Besides, Figure 2D shows a relatively small bias (−0.068 to 0.040) between estimated and assumed effects for each QTL in three simulation cases.

#### Effect of Sample Size on Mapping QTL

**FIGURE 3:**
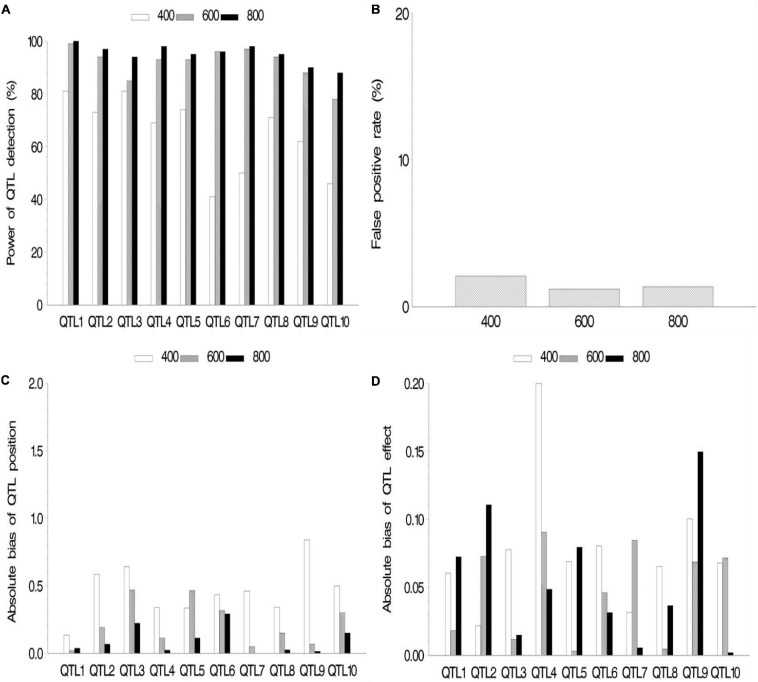
Effect of sample size on the new method. **(A)** Power of quantitative trait locus (QTL) detection, **(B)** false positive rate, **(C)** average of absolute bias between estimated and true positions, and **(D)** average of absolute bias between estimated and true effects.

In the second simulation, we investigated the effect of sample size on mapping QTL. The sample sizes were set as 400, 600, and 800 (*k* subpopulations each with 100 RILs), all the QTL sizes were set as 0.07, and other parameters were the same as those in the first simulation. The results are shown in Figure 3 and Supplementary Table 2.2. The results indicated the increase in statistical power in QTL detection and accuracy in QTL position estimation with the increase in sample size. FPR still stays on a low level (<2.1%). The effect estimates in this simulation showed more bias than those in the first simulation. This is possibly caused by smaller sample (400, 600, and 800) than those in the first simulation (964).

#### Random Effect Simulation

**FIGURE 4:**
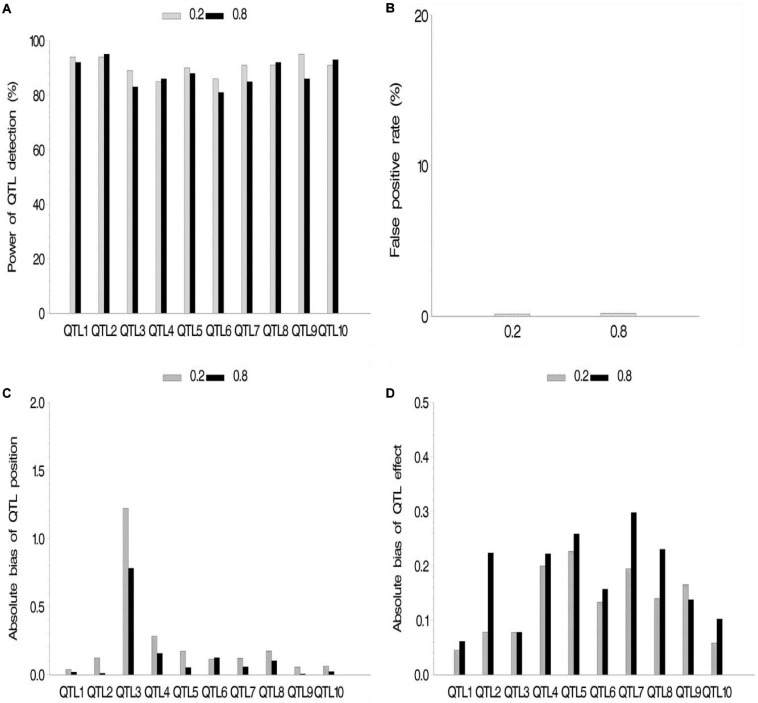
Effect of quantitative trait locus (QTL) type on the new method. **(A)** Power of QTL detection, **(B)** false positive rate, **(C)** average of absolute bias between estimated and true positions, and **(D)** average of absolute bias between estimated and true effects.

We also conducted a simulation experiment to investigate how fixed or random effect of QTL would influence our association mapping. A fixed effect was assigned to the first five QTLs, and there were no differences for these QTLs across various subpopulations, while random effects were assigned to the last five QTLs, and there were various values of *ρ* across various subpopulations. As a result, no significant difference between fixed and random effects in fixed *ρ* value was observed (Figure 4 and Supplementary Table 2.3), although their powers were more than 80%. Meanwhile, no significant difference among various *ρ* values in the same setup (fixed or random) of QTL effect was observed. FPR and the bias of QTL position and effect stayed a quite low level.

### Mapping QTLs for DA, DS, and ASI in Joint Maize NAM Subpopulations

The new method was used to identify QTLs for three flowering time traits in the joint maize NAM subpopulations. As a result, 77, 79, and 75 QTLs were identified, and these QTLs accounted for 90.11, 89.44, and 82.50% of the total phenotypic variances for the above three traits, respectively. Most QTLs detected by the CIM method in the single-maize NAM subpopulation were also identified by the new method in the joint maize NAM subpopulations. As for DA, the new results covered 127 of 137 QTLs from the CIM method (Supplementary Figure 1), including all the seven extremely large QTLs (*r*^2^ > 15%, light blue), 24 of 25 large QTLs (10% < *r*^2^ < 15%, deep green), 56 of 59 relative large QTLs (5% < *r*^2^ < 10%, deep blue), and 40 of 46 small QTLs (2% < *r*^2^ < 5%, pink) (Supplementary Figure 1). As for DS, 132 of 138 QTLs from the CIM method were covered by our new method, including all the five extremely large QTLs, 21 of 23 large QTLs, 71 of 75 relative large QTLs, and all the 35 small QTLs. As for ASI, 81 of 89 QTLs from the CIM method were found by the new method, including 21 of 22 large QTLs, 55 of 62 relative large QTLs, and all the 5 small QTLs. Clearly, the above results validated our new method.

As compared with the CIM results, we detected 25 additional QTLs for DA, 29 for DS, and 32 for ASI (Supplementary Table 3). The genetic variances of all additional QTLs were quite small. Most QTL for DA and DS accounted for < 1% phenotypic variance by a single QTL, although 8 of 32 QTLs for ASI accounted for more than 1% by a single QTL, and 5 QTLs accounted for more than 3% by a single QTL. This indicated that our new method had a high power for detecting minor alleles.

To validate these additional QTLs, we mined candidate genes around the above additional QTLs via phytozome v9.1.^3^ All the additional QTLs were found to be very close to their candidate genes, and these candidate genes were listed in Supplementary Table 3. For example, 19 of 25 candidate genes for DA, as well as 21 of 29 candidate genes for DS, were found to be within the distance of 1 kb from their associated SNPs. Among candidate genes for ASI, 23 of 31 genes were within 1 kb, and only two genes were found to be within >5 kb. The close distance indicated a strong linkage between associated SNPs and their candidate genes. Some evidence for candidate genes were described as below (Supplementary Table 3). GRMZM2G154896, near the SNP PZA00368.1 associated with DA, is a pollen tube developmental gene; GRMZM2G177151, near the SNP associated with DS, is C2H2-type zinc finger protein gene, which may play an important role in spike development; and GRMZM2G061900, near the SNP PZA00276.18 associated with ASI, is Ras protein gene that affects cell growth, differentiation, cytoskeleton, protein transport, and secretion.

## Discussion

Compared with QTL mapping in biparental segregation population, multiparental population could provide high power and resolution for association mapping in the genetic dissection of complex traits. This is because the association mapping population has more historical recombination events and high linkage disequilibrium (LD), which can increase allelic diversity and mapping resolution. However, conventional association mapping is always confounded by population structure between diverse lines (Flint-Garcia et al., 2005; Yu et al., 2006). The NAM design promised to address these weaknesses and utilize the advantages of linkage and association mapping (Yu et al., 2008). Therefore, it is necessary to propose an optimal approach in the genetic analysis of complex traits in the NAM population.

In this study, we found that genetic heterogeneity was a common factor in the NAM population, which would confound the results of association mapping. Thus, we proposed a multi-locus association model for mapping QTL of complex trait in the NAM population. This model could discriminate the QTL effects across various subpopulations, which addressed the problem of genetic heterogeneity across subpopulations. Because of “p≫ n” in the new model, we proposed a matrix transform approach to shrink the information of independence indicator variables. A multi-locus mapping method, involving with E-Bayes (Xu and Jia, 2007: Xu, 2010) and LRT, were proposed in this study.

In genetic analysis of the NAM population, jointing all families as mapping population is more common than using a single family, such as joint linkage mapping (JLM; Buckler et al., 2009; Tian et al., 2011), JICIM (Li et al., 2011), NAM (Xavier et al., 2015), and GWAS with mixed linear model (Chen et al., 2019). Because it had higher mean prediction ability and performed better at more stringent significance threshold. However, Li et al. (2011) observed that joint multifamily analysis has less power and worse resolution than single family for rare QTL, which is identified in only one or few subpopulations. Ogut et al. (2015) showed that most robust QTLs were restricted to one family and were often not detected at high frequency by joint family analysis. In this study, we found that most rare QTLs with large effect can be detected by our new method. For three flowering time traits, we can detect more than 90% of QTLs from the CIM method in the single NAM subpopulation. Besides, the new method can identify more small-effect QTLs than the CIM method.

In order to compare single family analysis (SF) with our new method, we conducted a series of simulations (more details about the simulation and results, see Supporting Information S4). In the simulation, 10 QTLs with five types of effects were assumed across five subpopulations. Stepwise regression was used for SF analysis, described by Buckler et al. (2009). The results showed that SF stepwise regression was powerful for large-effect QTLs rather than small-effect QTLs in the single-family NAM subpopulation. Because there are much less lines in the single-family NAM subpopulation than in the joint multifamily NAM subpopulations, enough precision for QTL detection cannot provided. However, our new method with multiple families had good power, not only for large- and small-effect QTLs but also for common and rare QTLs. On the one hand, joint multiple families increased population size (usually more than 20 times according to the NAM design) (Li et al., 2011). On the other hand, the new NAM model could discriminate QTL effects across various subpopulations, which controlled false positive signals from sample variance in nonrelated families. In addition, multi-locus GWAS methods are more powerful and robust in the detection of small-effect QTNs (Wang et al., 2016; Su et al., 2018; Wen et al., 2018; Zhang et al., 2019).

Some GWAS software packages are available in the NAM population, such as Trait Analysis by Association, Evolution, and Linkage (TASSEL; Chen et al., 2019) and Jawamix5 (Long et al., 2013). With TASSEL, MLM method can capture the population structure and genetic relatedness of all the lines in the NAM population by Q and K matrices. Jawamix5 also provides a fast GWAS tool in structured populations using the mixed model, as well as stepwise regression in NAM design (Long et al., 2013). These GWAS software packages are very powerful in normal GWAS. However, they were not designed for the NAM population and did not involve the genetic heterogeneity. We have proved that genetic heterogeneity from parents contributed to the diverse effects of a QTL in different families (Supplementary Table 1 and Figure 1). Therefore, the proper mapping methods are important, especially for the NAM population.

Joint linkage mapping (Buckler et al., 2009) and JICIM (Li et al., 2011) used the stepwise linear regression and linkage mapping to select marker effects nested within families and estimate QTL effects. It might lead to missing some large-effect QTLs identified only in one subpopulation (Ogut et al., 2015). In the NAM software (Xavier et al., 2015), a mixed linear model framework with EMMA algorithm (Kang et al., 2008) was used to map associated SNPs in multiparent population, such as the MAGIC population. Recently, this software was also used to detect QTLs in the NAM population (Sunil et al., 2020). In the genetic model of NAM software, the dimension will inflate to *k*+ 1 times (*k* families in NAM design), although marker effects can be estimated.

Our new method was designed for association mapping in the NAM population. Based on Monte Carlo simulation experiments and real data analysis, some minor QTLs can be identified by the new method, indicating high QTL signal to noise ratio in the NAM mapping population. The new method gave a dimension reduction via matrix transformation, which can maintain the family information in the genetic model and reduce computational burden. Actually, this approach could be applied in genome-wide association studies (Lü et al., 2011). In this study, the new method was validated in the NAM mapping population. However, it is also suitable for MAGIC population, which is a large RIL population derived from multiple parents (Cavanagh et al., 2008). Thus, the new method is useful in genetic mating design.

## Data Availability Statement

The author selected the following statement: Publicly available datasets were analyzed in this study. This data can be found here: http://www.panzea.org (Buckler_etal_2009_Science_flowering_time_data-090807.zip).

## Author Contributions

SB performed the experiments, analyzed the data, and drafted the manuscript. WW and Y-MZ discussed the results. All authors designed and conceived the experiments.

## Conflict of Interest

The authors declare that the research was conducted in the absence of any commercial or financial relationships that could be construed as a potential conflict of interest.

## Footnotes

1 http://www.panzea.org

2 https://brcwebportal.cos.ncsu.edu/qtlcart/WQTLCart.htm

3 http://www.phytozome.net/

## Supplementary Material

Supplementary Figure 1 Comparison of the new method in joint multi-family NAM sub-populations with the CIM method in single family for days to anthesis (DA).
