# Supplementary material for: A Multi-Locus Association Model Framework for Nested Association Mapping With Discriminating QTL Effects in Various Subpopulations
Source: Front Genet. 2021 Jan 18;11:590012. doi: 10.3389/fgene.2020.590012 (PMC7848182; doi:10.3389/fgene.2020.590012)
Supplement: Supplementary Figure 1 — Comparison of the new method in joint multi-family NAM sub-populations with the CIM method in single family for days to anthesis (DA). [file Image_1.pdf]

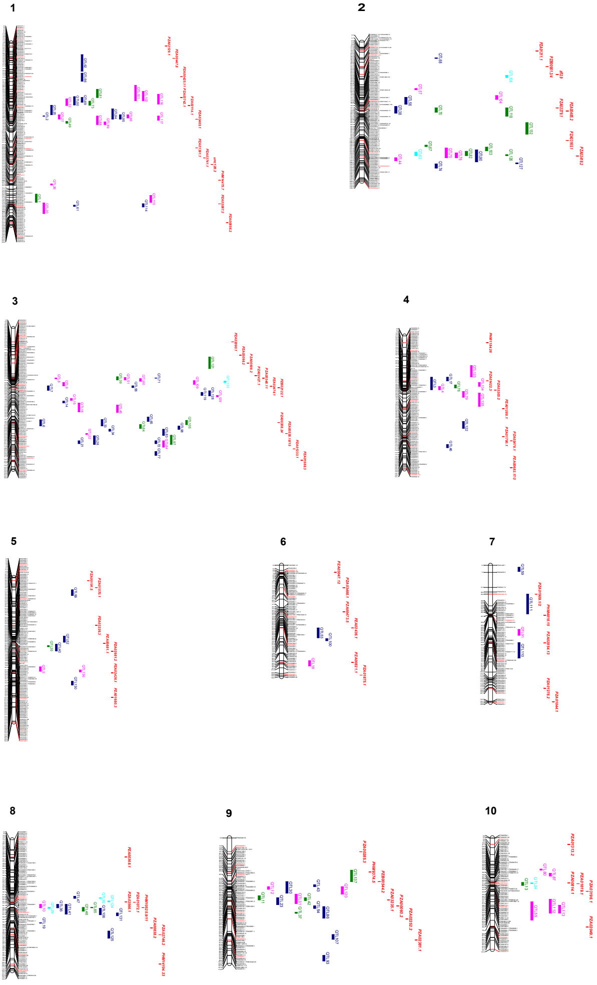

Supplementary Figure 1 Comparison between joint-family new method and single-family CIM for days to anthesis (DA)
