# Supplementary material for: A Multi-Locus Association Model Framework for Nested Association Mapping With Discriminating QTL Effects in Various Subpopulations
Source: Front Genet. 2021 Jan 18;11:590012. doi: 10.3389/fgene.2020.590012 (PMC7848182; doi:10.3389/fgene.2020.590012)
Supplement: Supplementary file 5 [file Table_4.DOCX]

Supporting Information S4

**The simulation for comparing Single Family Stepwise regression and the new method with multiple families**

We simulated 10 assumed QTLs with 5 type of effects in this section. The mapping population and positions of assumed QTLs were followed the former simulation in the manuscript. The heritability was set to 0.5. Each of QTLs contributed 5% of phenotypic variance in general population. According the equations of genetic variance and genetic effect above, we conducted the reference genetic effects (*α*) of all QTLs. In order to simulate different kinds of QTLs, we setted different QTL effects across different families (Table 1). Therefore, common QTL (exists in most of sub-populations) and rare QTL (exists only in one or few sub-populations) were both involved in this simulation.

The results (Table 2) showed that our new method had good power for all kinds of QTLs, especially the common QTL (QTL1 and QTL2). For rare QTL (QTL7, QTL8), the detecting power were relatively low. Single-Family Stepwise Regression could be powerful for large-effect QTLs. For instance, the former 4 QTLs in SF1 and SF5, QTL5 and QTL6 in SF3, QTL7 and QTL8 in SF1, QTL9 and QTL10 in SF5, all had relatively high power. Nevertheless，the other small effects were hard to detected.

**Table 1 The True Effects of 10 Assumed QTL across 5 Families**

| **Family** | **QTL1** | **QTL2** | **QTL3** | **QTL4** | **QTL5** | **QTL6** | **QTL7** | **QTL8** | **QTL9** | **QTL10** |
| --- | --- | --- | --- | --- | --- | --- | --- | --- | --- | --- |
| **1** | -1.41 | -1.41 | -1.41 | -1.41 | 0.00 | 0.00 | -1.41 | -1.41 | 0.00 | 0.00 |
| **2** | -0.71 | -0.71 | 0.00 | 0.00 | -0.71 | -0.71 | 0.00 | 0.00 | 0.00 | 0.00 |
| **3** | 0.00 | 0.00 | 0.00 | 0.00 | 1.41 | 1.41 | 0.00 | 0.00 | 0.00 | 0.00 |
| **4** | 0.71 | 0.71 | 0.00 | 0.00 | 0.71 | 0.71 | 0.00 | 0.00 | 0.71 | 0.71 |
| **5** | 1.41 | 1.41 | 1.41 | 1.41 | 0.00 | 0.00 | 0.00 | 0.00 | 1.41 | 1.41 |

**Table 2 The Power (%) of New Method and SF Stepwise Regression**

| **Method** | **QTL1** | **QTL2** | **QTL3** | **QTL4** | **QTL5** | **QTL6** | **QTL7** | **QTL8** | **QTL9** | **QTL10** |
| --- | --- | --- | --- | --- | --- | --- | --- | --- | --- | --- |
| **New** | 94 | 82 | 89 | 80 | 57 | 68 | 47 | 32 | 61 | 85 |
| **SF1** | 100 | 91 | 81 | 72 | 0 | 1 | 45 | 50 | 0 | 1 |
| **SF2** | 3 | 0 | 0 | 0 | 1 | 3 | 2 | 0 | 0 | 2 |
| **SF3** | 1 | 2 | 0 | 2 | 94 | 77 | 1 | 2 | 0 | 0 |
| **SF4** | 12 | 6 | 0 | 0 | 31 | 1 | 1 | 0 | 43 | 20 |
| **SF5** | 100 | 90 | 56 | 94 | 0 | 1 | 0 | 1 | 98 | 74 |

SF1– SF5, Single-Family Stepwise Regression in corresponding family
