# Supplementary material for: A Multi-Locus Association Model Framework for Nested Association Mapping With Discriminating QTL Effects in Various Subpopulations
Source: Front Genet. 2021 Jan 18;11:590012. doi: 10.3389/fgene.2020.590012 (PMC7848182; doi:10.3389/fgene.2020.590012)
Supplement: Supplementary file 6 [file Table_5.DOCX]

Supporting Information S5

**The codes of the new nested association mapping method in SAS**

infile aa dlm=',' lrecl=1000000 firstobs=2;

input pop x1-x100;

run;

data pheno;

infile bb dlm=',' lrecl=1000000 firstobs=1;

input y1-y100;

run;

proc iml;

symsize=90000000;

use geno;read all into geno;close geno;

use pheno;read all into pheno;close pheno;

start ebayes_EM(x,z,y,aaa);

n=nrow(z); k=ncol(z);

b=(x`*x)**-1*(x`*y);

v0=(y-x*b)`*(y-x*b)/n;

u=j(k,1,0);v=j(k,1,0);s=j(k,1,0);

do i=1 to k;

zz=z[,i];

s[i]=(zz`*zz)**-1*v0; * The variance of QTL effect var(gamma);

u[i]=s[i]*zz`*(y-x*b)/v0; * The expectation of QTL effect E(gamma);

v[i]=u[i]**2+s[i]; * The prior variance of QTL effect sigma(i);

end;

vv=j(n,n,0);do i=1 to k;zz=z[,i];vv=vv+zz*zz`*v[i];end;vv=vv+i(n)*v0;

iter=0; err=1000; iter_max=100; err_max=1e-8;

tau=0; * tau is the prior belief of the scaled inverse chi-square;

omega=0; * omega is the scale of the inverse chi-square;

do while (iter<iter_max & err>err_max);

iter = iter+1;

v01=v0;v1=v;b1=b;

vi=vv**-1;b=inv(x`*vi*x)*(x`*vi*y);r=y-x*b;

ss=j(n,1,0);

do i=1 to k;

zz=z[,i];

u[i]=v[i]*zz`*vi*r;

s[i]=v[i]*(1-zz`*vi*zz*v[i]);

v[i]=(u[i]**2+s[i]+omega)/(tau+2+1); * scaled inverse chi-square;

ss=ss + zz*u[i];

end;

v0=r`*(r-ss)/n;

vv=j(n,n,0);do i=1 to k;zz=z[,i];vv=vv+zz*zz`*v[i];end;vv=vv+i(n)*v0;

err=( (b1-b)`*(b1-b)+(v01-v0)**2+(v1-(v))`*(v1-(v)) )/(1+1+k);

beta=b`;

sigma2=v0;

end;

free uuu;

do i=1 to k;

stderr=sqrt(s[i]+ 1e-20);

t=u[i]/stderr;

if abs(t)>1.5 then uuu=uuu//(i||v[i]||u[i]||stderr||t);

else uuu=uuu//(i||v[i]||u[i]||stderr||.);

end;

mnv=1.00/sqrt(sum(v)/nrow(v)+0.0001);

aaa=b||uuu[,3]`||v0||mnv;

finish ebayes_EM;

pop=geno[,1];geno=geno[,2:ncol(geno)];z=geno;yy=pheno;pp=unique(pop);

n=nrow(pheno);nn=ncol(geno);np=ncol(pp);

popn=j(np,1,0);

do i=1 to np;

do j=1 to n;

if pop[j]=pp[i] then popn[i]=popn[i]+1;

end;

end;

xx=geno;

do i=1 to n;

do j=1 to nn;

if xx[i,j]=0 then xx[i,j]=2;

if xx[i,j]=-0.5 then xx[i,j]=-1;

if xx[i,j]=0.5 then xx[i,j]=1;

end;

end;

free bbb lll;

do rec=1 to ncol(yy);

y=yy[,rec];;free zz;

do ii=1 to nn;

x=xx[,ii];uu=j(np*3,1,0);z=j(n,1,.);aa=j(np*3,1,0);

do jj=1 to np;

mean=sum((pop=jj)#y)/popn[jj];

x1=(pop=jj)#x;

uu[jj*3-2]=sum((x1=1)#y)/sum((x1=1))-mean;

uu[jj*3-1]=sum((x1=2)#y)/sum((x1=2))-mean;

uu[jj*3]=sum((x1=-1)#y)/sum((x1=-1))-mean;

end;

max=max(uu);min=min(uu);ll=max-min;

do i=1 to np*3;

if uu[i]<=min+ll/6 then aa[i]=-5;

if uu[i]>min+ll/6 & uu[i]<=min+ll/3 then aa[i]=-3;

if uu[i]>min+ll/3 & uu[i]<=min+ll/2 then aa[i]=-1;

if uu[i]>min+ll/2 & uu[i]<=min+2*ll/3 then aa[i]=1;

if uu[i]>min+2*ll/3 & uu[i]<=min+5*ll/6 then aa[i]=3;

if uu[i]>min+5*ll/6 then aa[i]=5;

end;

do i=1 to n;

do j=1 to np;

if pop[i]=j & x[i]=1 then z[i]=aa[j*3-2];

if pop[i]=j & x[i]=2 then z[i]=aa[j*3-1];

if pop[i]=j & x[i]=-1 then z[i]=aa[j*3];

end;

end;

zz=zz||z;

end;

call ebayes_EM(j(n,1,1),zz,y,bb);

do ii=2 to ncol(bb)-2;

if bb[ii]<1e-4 then bb[ii]=.;

end;

free w;at=0;

do ii=1 to nn;

if abs(bb[ii+1])>1e-4 then do; w=w||ii;at=at+1; end;

end;

lod=j(1,1+nn,.);

if at>0.5 then ad=j(n,1,1)||zz[,w];else ad=j(n,1,1);

if min(abs(eigval(ad`*ad)))<1e-6 then bb0=inv(ad`*ad+i(ncol(ad))*0.01)*ad`*y;else bb0=inv(ad`*ad)*ad`*y;

vv0=(y-ad*bb0)`*(y-ad*bb0)/n;

ll1=sum(log(abs(pdf('normal',y,ad*bb0,sqrt(vv0)))));

sub=1:ncol(ad);

if at>0.5 then do;

ad1=zz[,w];

if abs(det(ad1`*ad1))<1e-6 then bb1=inv(ad1`*ad1+i(ncol(ad1))*0.01)*ad1`*y;

else bb1=inv(ad1`*ad1)*ad1`*y;

vv1=(y-ad1*bb1)`*(y-ad1*bb1)/n;

ll0=sum(log(abs(pdf('normal',y,ad1*bb1,sqrt(vv1)))));

lod[1]=-2.0*(ll0-ll1)/(2.0*log(10));

do ii=1 to at;

ij=setdif(sub,ii+1);ad1=ad[,ij];

if min(abs(eigval(ad1`*ad1)))<1e-6 then bb1=inv(ad1`*ad1+i(ncol(ad1))*0.01)*ad1`*y;

else bb1=inv(ad1`*ad1)*ad1`*y;

vv1=(y-ad1*bb1)`*(y-ad1*bb1)/n;

ll0=sum(log(abs(pdf('normal',y,ad1*bb1,sqrt(vv1)))));

lod[1+w[ii]]=-2.0*(ll0-ll1)/(2.0*log(10));

end;

end;

do i=1 to nn;

if lod[i+1]<3 then do;lod[i+1]=.;bb[i+1]=.;end;

end;

bbb=bbb//bb;lll=lll//lod;

free ww;at=0;

do ii=1 to nn;

if lod[ii+1]^=. then do;

x=geno[,ii];w=j(n,np,0);at=at+1;

do i=1 to n;do j=1 to np;if pop[i]=pp[j] then w[i,j]=x[i];end;end;

ww=ww||w;

end;

end;

wwx=j(n,1,1)||ww;

if min(abs(eigval(wwx`*wwx)))<1e-6

then bbx=inv(wwx`*wwx+i(ncol(wwx))*0.01)*wwx`*y;

else bbx=inv(wwx`*wwx)*wwx`*y;

ef=j(np,nn,.);k=1;

do ii=1 to nn;

if lod[ii+1]^=. then do;

ef[,ii]=bbx[(1+(k-1)*np+1):(1+k*np)];k=k+1;

end;

end;

eff=eff//ef;

end;

create bbb from bbb;append from bbb;close bbb;

create lll from lll;append from lll;close lll;

create eff from eff;append from eff;close eff;

quit;
